# Mechanisms of thermal treatment on two dominant copepod species in O_3_/BAC processing of drinking water

**DOI:** 10.1007/s10646-021-02392-8

**Published:** 2021-03-31

**Authors:** Wei Jiang, Sheng Dong, Fangfang Xu, Jing Chen, Chen Gong, Antai Wang, Zhangli Hu

**Affiliations:** 1 Guangdong Technology Research Center for Marine Algal Bioengineering, Longhua Innovation Institute for Biotechnology, College of Life Sciences and Oceanography, Shenzhen University, Shenzhen, 518060 P. R. China; 2 Shenzhen Key Laboratory of Marine Bioresource & Eco-environmental Science, Shenzhen University, Shenzhen, 518060 P. R. China

**Keywords:** Advanced treatment of drink water, O_3_/BAC processing, *Phyllodiaptomus tunguidus*, *Heliodiaptomus falxus*, Water flea control, Thermal treatment

## Abstract

*Phyllodiaptomus tunguidus* and *Heliodiaptomus falxus* are dominant copepods species in drinking water processing plants in southern China. With a potential penetration risk, the breeding and leakage of copepods are drawing more and more attention in recent years. The current study provided a thermal treatment method to control copepods and their eggs. Results showed that: (1) the immediate death rates of *P. tunguidus* and *H. falxus* after heated to 34–40 °C for 5 min are positively correlated to the treatment temperatures (*P* < 0.01), and all individuals of the both species were eliminated after heated at 40 °C for 5 min; (2) overall hatching rates of *P. tunguidus* eggs were negatively correlated with treatment temperatures (*P* < 0.01) between 39–45 °C, with zero percent hatched after treatment at 45 °C for 5 min; (3) hatching rates of *H. falxus* were negatively correlated with treatment temperatures (*P* < 0.01) between 37–41 °C, with no nauplii hatched when treated at 41 °C for 5 min; (4) paraffin section histological examination indicated that thermal treatment caused severe damage to internal organs and egg structure. Finally, based on the experimental data, the application of the thermal treatment method was discussed in ozonation combined with biological activated carbon (O_3_/BAC) processing of drink water treatment.

## Introduction

Copepods are dominant species in freshwater zooplankton communities worldwide. By predation of primary producers, and providing feed to higher trophic levels, copepods play a vital role of energy flow in water ecosystem (Xu 2018). Due to overfishing and eutrophication of freshwater bodies in recent years, copepods bloom in reservoirs and lakes, and even in source water of many districts. *Phyllodiaptomus tunguidus* and *Heliodiaptomus falxus* are dominant copepods in source water of southern China (Gong et al. 2013). Copepods cannot be completely removed by traditional drinking water treatment. Studies indicated that copepods can often infiltrate sedimentation and filtration tanks because of their high mobility, causing the proliferation of these species and reaching a density up to 600 individuals/L (Xu et al. 2007; Su et al. 2009). The solid chitinous carapace of copepods is highly resistant against chemicals used in disinfection process before delivery (Lin et al. 2014), even 5 times of current chlorine and ozone doses had no long-term inhibitory effect on oocysts development in finished water (Dhillon et al. 2010; Hardin et al. 2010). Since 1995, a series of *Cyclops* leakage in water supply network were reported in Harbin and Shijiazhuang, China (Cui et al. 2002; Xing et al. 2005).

Zooplankton organisms, including copepods, provide a natural protection mechanism used by waterborne pathogenic bacteria and protozoa (Bichai et al. 2008). There is a natural relationship between copepods and *Vibrio cholerae*, which is a high risky pathogen transmitted through drinking water (Hurst 2018). It was reported that bacteria of the genus *Vibrio* play an important role in chitin mineralization by bonding to the chitin and using it as an exclusive carbon and nitrogen source (Heidelberg et al. 2002). Also, as intermediate hosts of many pathogenic protozoa including *Dracunculus medinensis*, *Schistosome* and *Nematodes*, the copepods penetration to the network is seriously threatening water supply safety and public health (Su et al. 2009; Eberhard et al. 2015; Hopkins et al. 2018).

In our previous study, copepod population and dynamic in drinking water plants using advanced O_3_/BAC processing in southern China were monitored and proved that current procedures cannot eliminate copepods. Here, we report the thermal treatment effect using a relatively low temperature to control copepods, and provide an operable treatment for drinking water plants.

## Materials and methods

### Copepod isolation and culture

*Phyllodiaptomus tunguidus* and *Heliodiaptomus falxus* were collected by filtration with #13 screen (113 μm mesh) from BAC filter in water plants using O_3_/BAC processing. After isolation under a stereo microscope, copepods were placed in 5 L glass tanks supplied with culture medium, which comprised 0.147 g CaCl_2_, 0.840 g NaHCO_3_, 0.0075 g KCl, 0.0203 g MgCl_2_, 0.5 g yeast extract, and 10 ml of a log-phase cultured *Chlorella vulgaris* per liter sterilized water, and cultured at room temperature (25 ± 2 °C) under a 10:14 h light: dark cycle with an intensity of 250 ± 50 Lux in light period. Dissolved oxygen was supplied with aquarium pumps.

### Adult copepods thermal treatment

The field water temperature of plants using O_3_/BAC processing fluctuates between 13–30 °C in southern China, and 30 °C was used as the lowest temperature for thermal treatment. The experiment of adult copepods included 6 treatment groups (group A to F) with a temperature at 30, 32, 34, 36, 38, 40 °C respectively, and a control group at 25 °C. Water baths were used for treatment, and heated to preset temperatures of different groups. A 500 ml beaker was set in each bath and filled with 400 ml fresh copepod culture medium. When the temperature of medium stabilized at the preset point, 100 ± 20 copepods were transferred into each beaker using a 113 μm mesh for thermal treatment. 5 min later, the *P. tunguidus* were transferred to room temperature and the death rate was immediately counted under a stereo microscope. Thereafter, the heat-treated copepods were cultured at 25 ± 2 °C, and counted for death rate until 48 h. Each treatment was carried out in triplicate.

### Copepod eggs thermal treatment

Both *P. tunguidus* and *H. falxus* eggs were heated under different temperatures and the effects on hatching were analyzed. For *P. tunguidus*, thermal treatments at 39, 41, 43, and 45 °C (group a to d) were conducted, while thermal treatment at 37, 39 and 41 °C (group e to g) were arranged for *H. falxus*. The thermal treatment procedure was described previously in 2.2. Egg-bearing copepods were treated in the pre-heated beakers for 5 min. Then oocysts were transferred to 12-well plates for hatching using a stereo microscope. Each well was filled with 3 ml medium and cultured at 25 ± 2 °C. The oocysts were continuously examined until 48 h, and the number of hatched eggs was counted. Each treatment was repeated 3 times and 30 copepods were used each time.

### Microscopic examination and data analysis

Samples of the control and treated groups were examined, counted, and photographed using a MZ16 stereo microscope (Leica, German). Digital images were processed with Photoshop 7.0. Copepod death rates and hatching rates were calculated for different thermal treatment temperatures. Death rates (M) and culture time (T) were fitting by univariate linear equation:

$$M = {\mathrm{a}}T + {\mathrm{b}}$$

Where a and b were the slop and interception, respectively. Single factor ANOVA was used to determine the effect of temperature on death rates and hatchability.

### Paraffin sectioning

Adult copepods and eggs of both *P. tunguidus* and *H. falxus* were thermal treated for 5 min under 41, 43, 45 °C and a control temperature at 25 °C. After treatment, copepods and eggs were washed with distilled water, then fixed in 10% formaldehyde overnight. The next day, samples were dehydrated, embedded with paraffin, and sectioned at 6 μm thickness and stained with hematoxylin and eosin (H. E.).

## Results

### Thermal tolerance of copepods

**Fig. 1:**
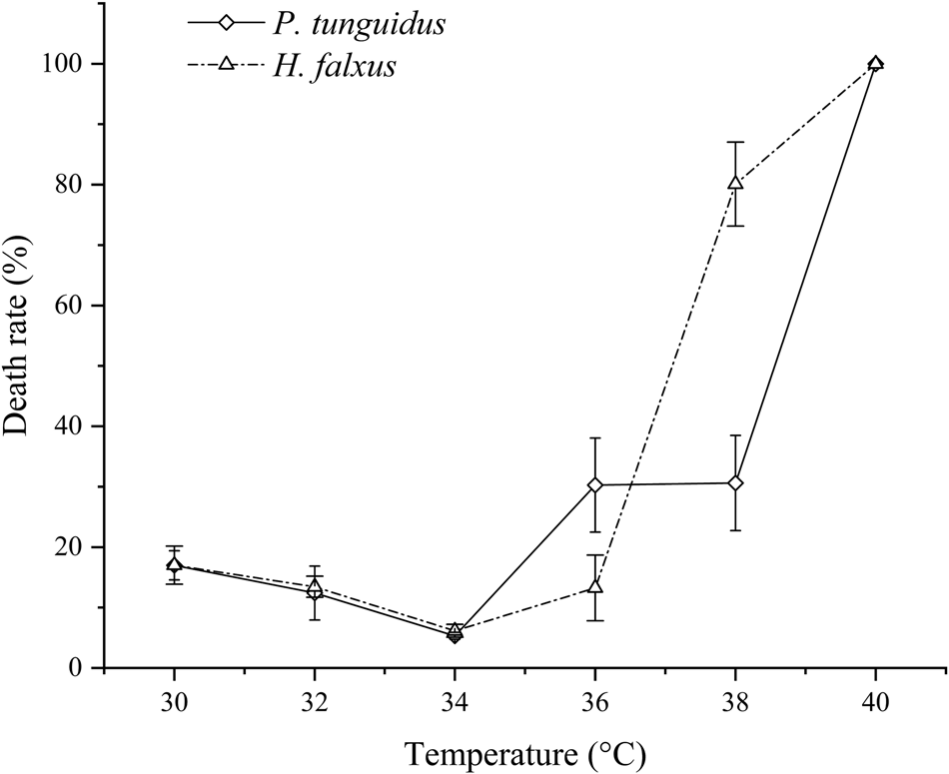
Death rates of two copepod species after heated for 5 min at different temperatures

The immediate death rates of *P. tunguidus* and *H. falxus* after 5 min thermal treatment at 30–34 °C both reduced. As the temperature further increased from 34 to 40 °C, the immediate death rates rise gradually (showed in Fig. 1). *P. tunguidus* and *H. falxus* also showed different sensitivity and tolerance to treated temperature. When the temperature increased from 34 to 36 °C, the death rate of *H. falxus* rose slower than *P. tunguidus*. After that, mortality of *H. falxus* increased rapidly and reached 80.01% at 38 °C. In contrast, only 30.61% of *P. tunguidus* died after a 38 °C treated, basically the same as that of 36 °C. After being heated at 40 °C for 5 min, all copepods of both species were exterminated (Fig. 1).

After a 5 min thermal treatment at different temperature, the death rate of *P. tunguidus* increased varying degrees comparing with control group. For the treatment groups A to F, 16.99, 12.40, 5.32, 30.27, 30.61 and 100.00% of *P. tunguidus* were eliminated, while only 3.56% copepods died in control group. At 34–40 °C, death rates of *P. tunguidus* were positively correlated with temperature (*P* < 0.01), and reached 100% at 40 °C. Also, for the control and treatment groups except group F, death rates of *P. tunguidus* increased gradually with follow-up culture time after thermal treatment. A linear regression analysis showed that the death rate in the control was correlated with culture time (*R*^2^ = 0.980). In contrast to the control group, the treatment groups showed weaker correlation, especially at higher temperatures (Table 1).

**Table 1:** Death rates of *P. tunguidus* at different follow-up culture time after different thermal treatment temperatures for 5 min

Group	Thermal treat temperature (°C)	Culture time after treatment (h)	Death rate (Mean(%) ± S.D.; *n* = 3)	Linear equation
Control	25	0	3.56 ± 3.43	*M* = 0.748 T + 4.1220 *R*^2^ = 0.980
		12	9.26 ± 3.14	
		24	17.23 ± 1.47	
		36	27.91 ± 5.20	
		48	35.21 ± 20.53	
A	30	0	16.99 ± 2.41	*M* = 0.544 T + 18.396 *R*^2^ = 0.941
		12	20.61 ± 1.32	
		24	32.63 ± 10.08	
		36	35.09 ± 5.34	
		48	39.42 ± 4.01	
B	32	0	12.41 ± 4.47	*M* = 0.719 T + 13.434 *R*^2^ = 0.952
		12	16.01 ± 2.31	
		24	30.40 ± 9.29	
		36	36.82 ± 8.46	
		48	41.27 ± 16.25	
C	34	0	5.32 ± 0.57	*M* = 0.837 T + 10.474 *R*^2^ = 0.827
		12	14.13 ± 5.04	
		24	36.33 ± 3.34	
		36	38.06 ± 6.42	
		48	39.71 ± 11.38	
D	36	0	30.27 ± 7.79	*M* = 0.391 T + 33.636 *R*^2^ = 0.873
		12	38.02 ± 1.90	
		24	43.70 ± 8.41	
		36	45.74 ± 1.72	
		48	48.36 ± 1.01	
E	38	0	30.61 ± 7.89	*M* = 0.343 T + 37.083 *R*^2^ = 0.615
		12	44.74 ± 7.19	
		24	46.14 ± 4.07	
		36	48.02 ± 7.78	
		48	49.14 ± 3.59	
F	40	0	100.00 ± 0.00	*M* = 100
		12	–	
		24	–	
		36	–	
		48	–	

### Hatchability of copepod eggs

**Fig. 2:**
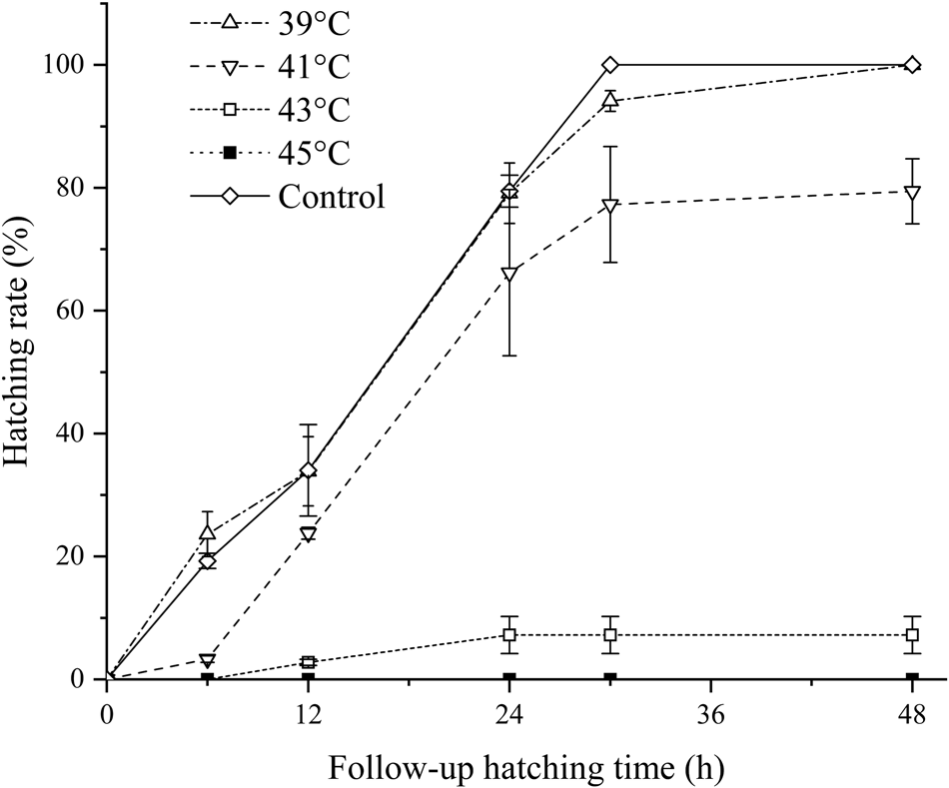
Hatching rate of *P. tunguidus* at different follow-up hatching time

The hatching rate was used as an indicator of heat tolerance for copepod eggs, and hatching results of both copepods were listed in Table 2. Results showed that all of *P. tunguidus* eggs in control group hatched within 30 h, while those in the treatment groups required longer hatching times with a lower hatching rate (Fig. 2). Treatment at 39 °C almost had no effect on hatching, and all eggs were able to hatch within 48 h. Higher temperatures partly inhibited copepod hatchability. After the treatment at 41 °C 79.44% eggs hatched within 48 h. The hatching rate decreased dramatically at 43 °C, and only 7.22% eggs hatched in 48 h. Treated at 45 °C, no nauplii hatched in 48 h. It was provided hatching rate was negatively correlated with the thermal treatment temperatures between 39–45 °C (*P* < 0.01). Over 92.78% *P. tunguidus* eggs were inactivated or inhibited under a 43 °C heat treatment, and no hatching observed at 45 °C.

**Table 2:** Hatching rates of two copepod species at different follow-up hatching time after different thermal treatment temperatures for 5 min

Copepod specie	Group	Thermal treat temperature (°C)	Follow-up hatching time (h)	Hatching rate (Mean(%) ± S.D.; *n* = 3)
*P. tunguidus*	Control	25	6	19.27 ± 1.23
			12	34.02 ± 7.44
			24	79.45 ± 2.61
			30	100.00 ± 0.00
			48	–
	a	39	6	23.64 ± 3.67
			12	33.86 ± 5.63
			24	79.12 ± 4.91
			30	94.12 ± 1.68
			48	100.00 ± 0.00
	b	41	6	3.30 ± 0.52
			12	23.78 ± 0.98
			24	66.18 ± 13.52
			30	77.28 ± 9.44
			48	79.44 ± 5.29
	c	43	6	0.00 ± 0.00
			12	2.77 ± 0.49
			24	7.22 ± 3.01
			30	7.22 ± 3.01
			48	7.22 ± 3.01
	d	45	6	0.00 ± 0.00
			12	0.00 ± 0.00
			24	0.00 ± 0.00
			30	0.00 ± 0.00
			48	0.00 ± 0.00
*H. falxus*	Control	25	6	19.65 ± 6.74
			12	100.00 ± 0.00
			30	–
			48	–
	e	37	6	0.00 ± 0.00
			12	67.03 ± 10.01
			30	77.84 ± 10.87
			48	100.00 ± 0.00
	f	39	6	0.00 ± 0.00
			12	29.19 ± 10.27
			30	54.05 ± 19.84
			48	65.00 ± 10.81
	g	41	6	0.00 ± 0.00
			12	0.00 ± 0.00
			30	0.00 ± 0.00
			48	0.00 ± 0.00

**Fig. 3:**
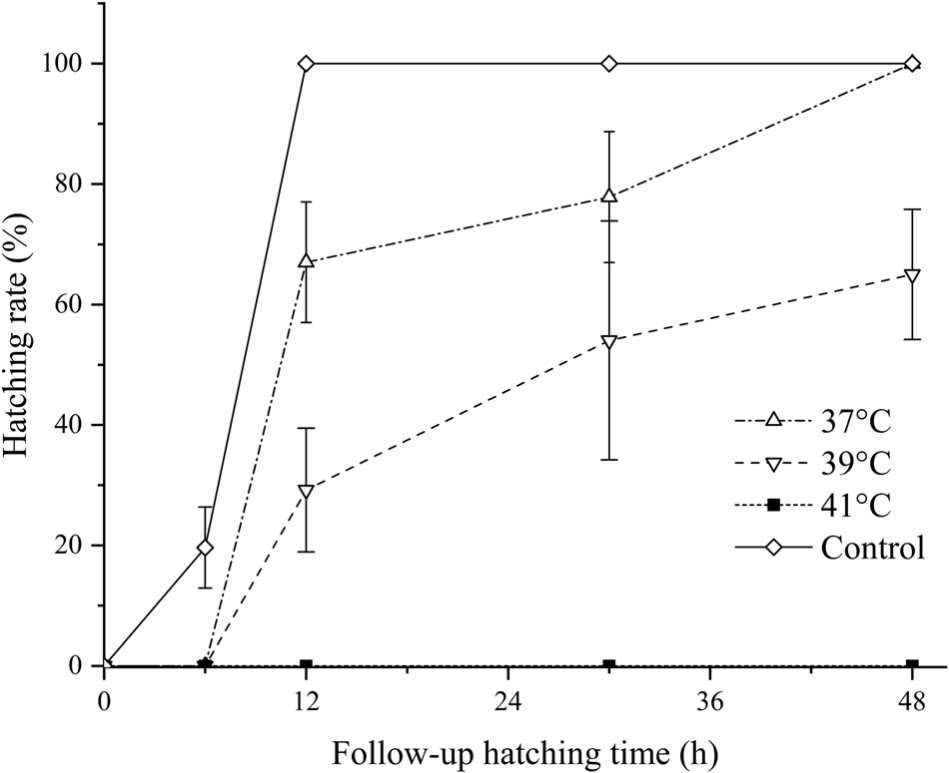
Hatching rate of *H. falxus* at different follow-up hatching time

The hatching rate of *H. falxus* eggs were showed in Fig. 3. All eggs in control group hatched within 12 h. Heated at 37 °C for 5 min had limited effect on hatching, and all eggs hatched within 48 h. A treatment at 39 °C for 5 min led to a reduction in the hatching rate to 65%. Heated at 41 °C for 5 min, no egg hatched within 48 h. There was a negative correlation between temperature within 37–41 °C and hatchability, where increases in temperature resulted in the reduction of the hatching rate (*P* < 0.01).

### Tissue structure changes in copepods and eggs

**Fig. 4:**
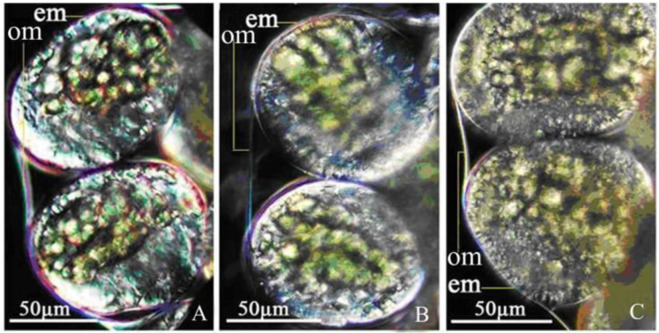
Copepod egg structure before and after heat treatment. **A** Eggs before heat treatment; (**B**) Egg structure after heated at 39 °C; (**C**) Egg structure after heated at 41 °C. em: egg membrane; om: oocyst membrane

Morphological results in Fig. 4 showed that thermal treatments caused significant overall structural changes. Normal eggs were completely encapsulated by an oocyst membrane. Both egg membrane and oocyst membrane were smooth. Eggs were transparent and displayed clear internal structures (Fig. 4A). After heated at 39 °C, the egg membrane and oocyst membrane became thinner, and the eggs became cloudy (Fig. 4B). After heated at 41 °C, oocyst membrane invaginated, fused with egg membrane, and egg membrane became rough even broken, and the egg became cloudy with denatured internal contents (Fig. 4C).

**Fig. 5:**
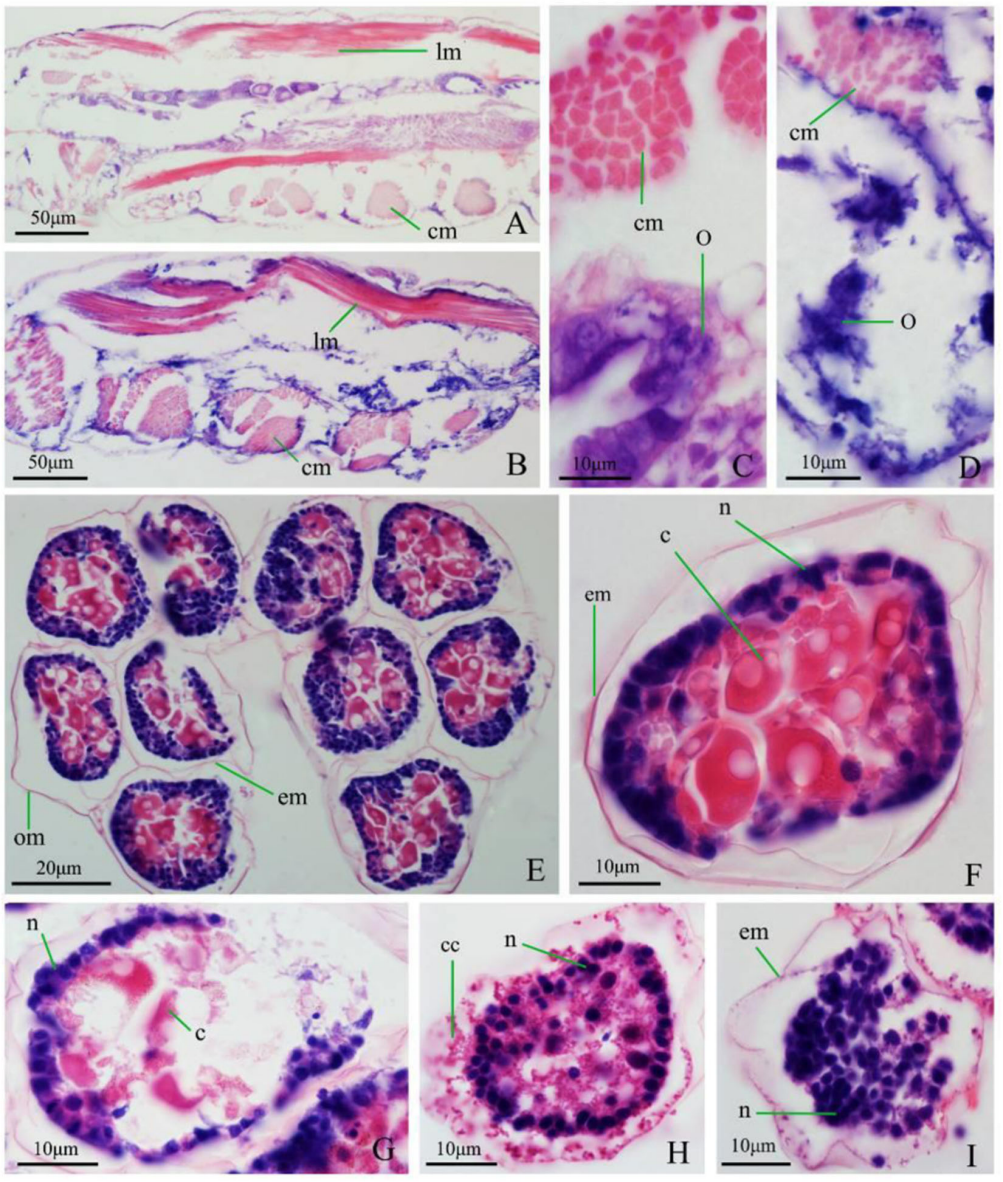
Paraffin section of copepods and copepod eggs. **A** Longitudinal section of head and thoracic segment in control group. **B** Longitudinal section of head and thoracic segment after thermal treatment at 45 °C for 5 min. **C** Longitudinal section of thoracic segment in control group. **D** Longitudinal section of thoracic segment after thermal treatment at 45 °C for 5 min. **E** Transverse section of oocysts in control group. **F** Transverse section of a single egg in control group. **G** Transverse section of a single egg after treated at 41 °C for 5 min. **H** Transverse section of a single egg after treated at 43 °C for 5 min. **I** Transverse section of a single egg treated at 45 °C for 5 min. *c* cytoplasm, *cc* cell contents, *cm* circular muscle, *em* egg membrane, *lm* longitudinal muscle, *n* nucleus, *O* ovary, *om* oocyst membrane

Also, there were clear differences in the tissue structure of copepods before and after the thermal treatment. The structure of control individuals was intact, with the body cavity completely enclosed by the carapace, circular and longitudinal muscle arranged tightly, no atrophy, and internal organs clearly visible and intact (Fig. 5A, C). After treatment, the copepods showed a damaged carapace between joints, broken and loosely arranged muscles, internal organs were unrecognizable, with only remnant left (Fig. 5B, D).

The thermal treatment caused severely damaged to egg structure. In the control group, eggs and oocysts showed intact structures. Eggs were tightly arranged (Fig. 5E), and nuclei arranged regularly along the perimeter and substantial cytoplasm (Fig. 5F). The treated oocysts were damaged, with broken membranes and scattered eggs. After heat treatment at 41 °C, the egg membrane was partially damaged, with leaked cytoplasm (Fig. 5G). After treatment at 43 °C, the egg membrane was severely damaged. Egg contents were denatured and exploded, and the nuclear structure was clearly damaged (Fig. 5H). After treatment at 45 °C, the egg disintegrated, and the egg membrane broke into pieces.

## Discussion

### Copepod control methods in drinking water treatment

Copepods are ubiquitous in source water, and can proliferate in granular filter of water treatment plants. As waterborne pathogens are noticed to maintain viability inside higher organisms, the proliferation and penetration of copepods might confer a remarkable risk to drinking water (Bichai et al. 2008). Extensively studied methods for copepods control were conducted including liquid chlorine, chlorine dioxide, chloramines, hydrogen peroxide, ozone, and combinations of these (Lin et al. 2010, 2012; Wang and Wang 2010). Chlorine is the most widely used drinking water disinfectant, but copepods have a strong resistance against chlorination and are not effectively inactivated by chlorination disinfection (Bichai et al. 2010). A combination of chlorine and chloramine disinfection method was reported, intracellular organic matter of *Cyclops* was well controlled under a 10 mg/L concentration of both chlorine and chloramine (Sun et al. 2016). Researchers suggested that chlorine dioxide had stronger effect than chlorine in general (Zhao et al. 2007; Lin et al. 2014; Nie et al. 2016). After 30 min contact, 100% of water fleas were inactivated at 1.0 mg/L chlorine dioxide, with the same contact time, the same inactivation rate required a concentration of 2.0 mg/L of liquid chlorine (Cui et al. 2003). Kos et al. (2020) also proved that chlorine dioxide was the most effective disinfectant, followed by ozone, while chlorine was the least efficient disinfectant in drinking water treatment. Cui et al. (2005) combined O_3_/H_2_O_2_ at various concentrations, and results showed that complete extermination of copepods could be achieved at 1.0 mg/L of O_3_ combined with 4 mg/L H_2_O_2_ with a 30 min contact.

However, it was found that ozonation treated BAC filter in water processing plants still had large amounts of copepods (Gong et al. 2013). Su (2007) suggested that copepod density and activity reduced step-by-step as treatments proceeding, but a dramatical increase of both copepod density and activity were detected in activated carbon filter compared with the upstream sand filter. The oxidation deactivation process in drinking water treatment was simulated, and it was found that copepods were eliminated under conventional concentration of oxidants used in water plants, but even using a triple conventional concentration, oxidants had limited effect on copepod eggs (to be published separately). Thus, common oxidants cannot inhibit copepod egg development. Advanced treatment such as O_3_/BAC processing, with a stable water condition, suitable temperature, sufficient dissolved oxygen, abundant microorganisms and organic matter, provides ideal condition for copepod proliferation. In the BAC filter, there is a risk that the infiltrated eggs hatch, develop and grow to adult, forming new populations of copepods. Oxidants can only eliminate hatched copepod individuals, but cannot prevent secondary outbreaks.

The current study explored using a heating method to deactivate copepods. Results showed that thermal treatment at 40 °C for 5 min was able to eliminate all copepods, and that heat to 45 °C for 5 min could completely deactivate copepod eggs (Figs. 1–3). The approach required much less time than oxidant treatment. Not only can it completely eliminate copepods but also their eggs, therefore prevents secondary proliferation of copepods in O_3_/BAC processing.

### Mechanism of thermal lethality in drinking water treatment

Temperature is the most important factor for the growth of zooplankton individuals and development of zooplankton communities, and plays a key role for copepod productivity (Alba et al. 2019; Zhang et al. 2017). Copepod physiological activities are profoundly affected by temperature fluctuations. Jiang et al. (2009) showed that (1) a rapid increase of 5 °C (to 25 °C) negatively affected the growth of copepods, (2) a rapid increase of 8 °C (to 28 °C) reduced copepod survival rate and (3) copepod mortality was significantly increased at 32 °C. When approaching the lethal temperature, copepods showed a reduced metabolism, energy consumption, and heat tolerance, ultimately leading to death (Jiang et al. 2010). Zeng et al. (2009) indicated that copepods had a certain level of adaptability for temperature changes. Exceeding the limits would cause injury and death.

In this study, we showed that the copepod survival rate decreased (*P* < 0.01) when the water temperature rose to above 34 °C. All copepods were killed at 40 °C. Experiments showed that copepod heat tolerance was slightly increased when the water temperature rose, but there was a heat tolerance limit. The current study showed that both copepod species cannot survive 40 °C. At such a temperature, copepods were apparently damaged, displaying broken and separated muscle fiber, fuzzy internal organ and only skeletal remnants remained (Fig. 5). Further detailed studies are needed to determine whether other species can tolerate 40 °C.

### Inhibition of hatching by thermal treatment

Devi and Gupta (2017) believed that temperature played a vital factor for the zooplankton growth. It was showed that temperature had effects on copepod *Acartia tonsa*, and the egg hatching rates, development, female sizes, and egg sizes would decrease under an improper temperature (Hansen et al. 2016). The current study showed that the treatment at 39 °C for 5 min resulted in a higher hatching rate within 12 h, compared with the control. The treatments at 40–44 °C resulted in the decreased hatching rates. Hatching was completely inhibited at 45 °C. As the treatment temperature increased, egg membrane became thinner, damaged to various extents. At 45 °C, egg membrane ruptured as the contents overly expanded, resulted in content spills and basic structure damages.

In summary, thermal treatment at 45 °C not only eliminate all copepods, but also completely inhibits the development of eggs. A heat treatment in drinking water processing can prevent secondary chemical contamination and not produce any byproducts. It is also advantageous in energy saving, and has less effect on active bacterial community in the BAC filter. Still, the result that hatching rate of *P. tunguidus* increased slightly when cultured 6 h after a 39 °C treatment, needs further investigation.

### Applicability of thermal treatment in water processing

Common methods to control water fleas include applications of chlorine dioxide, liquid chlorine and ozone. Drawbacks of these methods are obvious. (1) Chlorine dioxide emits toxic chlorite and chlorate. The preparation and application of chlorine dioxide require complicate operations. High purity products are expensive. There are safety concerns during transportation and storage of chlorine dioxide. Therefore, the application of the chlorine dioxide in drinking water treatment is limited (Wang and Liu 2005). (2) Chlorine can efficiently exterminate water fleas by reacting with organic and inorganic materials, but it generates unhealthy disinfection by-products (DBPs) including trichlormethane and Haloacetic acids (Sun et al. 2013; Mckie et al. 2015). (3) O_3_ as a strong oxidant, combined with activated carbon, has been widely used in advanced water treatment. Considering ozone breaks down rapidly in water, it is difficult to maintain its activity. All the by-products are hydrophilic, and some of these as formaldehyde and acetaldehyde are carcinogenic or potentially carcinogenic (Papageorgiou et al. 2014). In addition, since water fleas can have strong antioxidation capability, they can survive, penetrate, and proliferate in sedimentation tanks. Adult copepods may be enriched in filtration materials, blocking water flow. Nauplii and eggs may infiltrate filters and enter pure water tanks, and finally enters the pipeline (Xing et al. 2005).

The heating method with proper temperature can destroy the structure of copepods tissue and their eggs and eliminate them completely in a short time. This treatment is simple and highly applicable in drinking water processing, without by-products and secondary pollution. In field drinking water plants, the combination with high temperature steam or cooling water from other industry as electric plants, can further save energy and costs. Practically, before back washing of activated carbon filter, a proper proportion of high temperature cooling water from electricity plants can be injected to reach 45 °C and maintained for 10–15 min. This method can completely exterminate copepod including *P. tunguidus* and *H. falxus* and their eggs. It should be noted that the method has not been tested in drinking water treatment. Since there are seasonal temperature fluctuations, it is necessary to monitor water temperatures in filters, and set cooling towers to ensure a proper treatment temperature.
